# New Permian fauna from tropical Gondwana

**DOI:** 10.1038/ncomms9676

**Published:** 2015-11-05

**Authors:** Juan C. Cisneros, Claudia Marsicano, Kenneth D. Angielczyk, Roger M. H. Smith, Martha Richter, Jörg Fröbisch, Christian F. Kammerer, Rudyard W. Sadleir

**Affiliations:** 1 Centro de Ciências da Natureza, Universidade Federal do Piauí, 64049-550 Teresina, Brazil; 2 Programa de Pós-Graduação em Geociências, Departamento de Geologia, Universidade Federal de Pernambuco, 50740-533 Recife, Brazil; 3 Departamento de Cs. Geologicas, FCEN, Universidad de Buenos Aires, IDEAN-CONICET, C1428EHA Ciudad Autónoma de Buenos Aires, Argentina; 4 Integrative Research Center, Field Museum of Natural History, 1400 South Lake Shore Drive, Chicago, Illinois 60605, USA; 5 Department of Karoo PalaeontologyIziko, South African Museum, PO Box 61, Cape Town 8000, South Africa; 6 Evolutionary Studies Institute, University of Witwatersrand, Private Bag 3, 2050 Johannesburg, South Africa; 7 Earth Sciences Department, Natural History Museum, Cromwell Road, London SW7 5BD, UK; 8 Museum für Naturkunde, Leibniz−Institut für Evolutions- und Biodiversitätsforschung, Invalidenstr. 43, D−10115 Berlin, Germany; 9 Institut für Biologie, Humboldt-Universität zu Berlin, Invalidenstr. 110, D−10115 Berlin, Germany; 10 Department of Biological Sciences, Saint Xavier University, 3700 West 103rd Street, Chicago, Illinois 60655, USA

## Abstract

Terrestrial vertebrates are first known to colonize high-latitude regions during the middle Permian (Guadalupian) about 270 million years ago, following the Pennsylvanian Gondwanan continental glaciation. However, despite over 150 years of study in these areas, the biogeographic origins of these rich communities of land-dwelling vertebrates remain obscure. Here we report on a new early Permian continental tetrapod fauna from South America in tropical Western Gondwana that sheds new light on patterns of tetrapod distribution. Northeastern Brazil hosted an extensive lacustrine system inhabited by a unique community of temnospondyl amphibians and reptiles that considerably expand the known temporal and geographic ranges of key subgroups. Our findings demonstrate that tetrapod groups common in later Permian and Triassic temperate communities were already present in tropical Gondwana by the early Permian (Cisuralian). This new fauna constitutes a new biogeographic province with North American affinities and clearly demonstrates that tetrapod dispersal into Gondwana was already underway at the beginning of the Permian.

## Abstract (web summary)

The biogeographic origins of Permian terrestrial vertebrates in high-latitude regions remain poorly understood. Here, the authors report an early Permian continental tetrapod fauna from South America in tropical Western Gondwana that constitutes a new biogeographic province with North American affinities.

## Graphical abstract

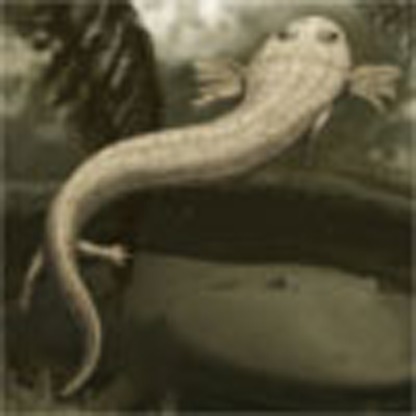

The Cisuralian (early Permian, *ca*. 298–272 million years ago) record of continental tetrapods in Gondwana has long been elusive. The iconic marine mesosaurs from Africa and South America are the only well-known Cisuralian Gondwanan tetrapods^1^,^2^. However, trackways from Argentina^3^ and fragmentary body fossils from Namibia^4^ hint at a greater tetrapod diversity at the time. Altogether, these records indicate that Gondwanan tetrapods had adapted to both salt-water and fully terrestrial environments, and achieved a broad geographic distribution, implying a deep history of tetrapod communities in the southern hemisphere^3^.

The Parnaíba Basin of northeastern Brazil hosts the Pedra de Fogo (PFF) and Motuca (MF) formations, which have produced a wealth of plant and fish fossils, as well as tantalizing remains of the large temnospondyl amphibian *Prionosuchus plummeri*^5^ from the uppermost PFF. The age of these units is considered Kungurian (latest Cisuralian) based on biostratigraphic correlations with the Chemnitz Fossil Forest of Germany and the Irati Formation of southern Brazil, which both have radiometric dates of 278 million years ago^6^,^7^,^8^,^9^. Despite the proven potential, the tetrapod fauna of the PFF has received almost no attention from vertebrate palaeontologists in the past half century. The new vertebrates from the lower PFF lacustrine beds reported herein provide an unparalleled window into tropical wetland faunas of Gondwana during the Cisuralian.

## Results

### Systematic palaeontology

       Temnospondyli Zittel, 1888

     Dvinosauria Yates & Warren, 2000

**Figure 1:**
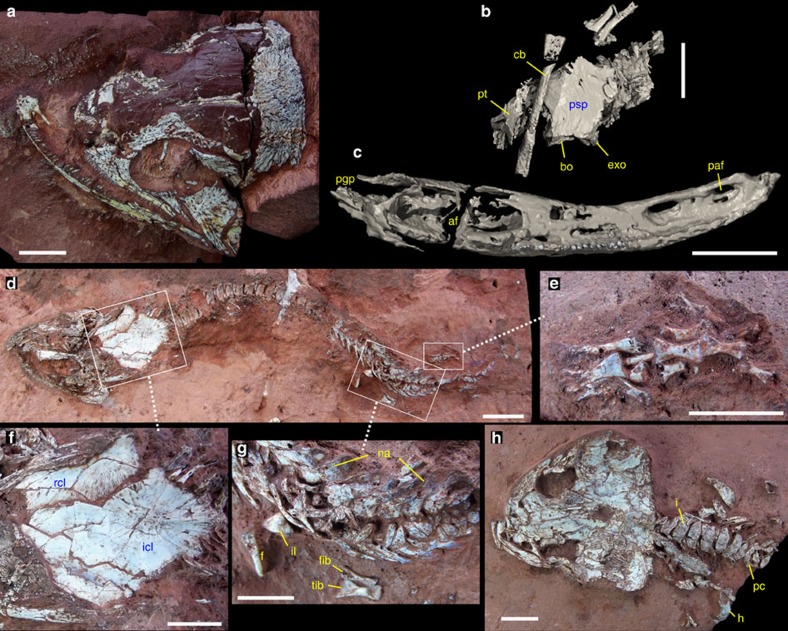
The dvinosaur temnospondyl *Timonya anneae* gen. et sp. nov. from the Cisuralian of northeastern Brazil. (**a**–**c**) holotype UFPI PV001. (**a**) dorsal view of skull. (**b**) CT rendering of posterior portion of palate in ventral view. (**c**) CT rendering of right jaw, occlusal view. (**d**–**h**) articulated skeleton of a juvenile individual in dorsal view, UFPI PV004. (**d**) general view of the skeleton. (**e**) details of the right pes. (**f**) pectoral girdle. (**g**) pelvic girdle and left limb. (**h**) counter-slab of UFPI PV004 showing skull roof in ventral view and details of anterior thoracic vertebrae and right forelimb. af, adductor fossa; bo, basioccipital; cb, ceratobranchial; exo, exoccipital; f femur; fib, fibula; h, humerus; i, intercentrum; icl, interclavicle; il, ilium; na, neural arches; paf parasymphysial foramen; pc, pleurocentrum; pgp, postglenoid process of the jaw; psp, parasphenoid; pt, pterygoid; rcl, right clavicle; tib, tibia. Scale bar, 10 mm (**a**–**d**) and 5 mm (**e**–**h**).

      *Timonya anneae* gen. et sp. nov.

  (Fig. 1, Supplementary Fig. 1, Supplementary Movie 1)

 **Etymology.** *Timonya* refers to Timon Municipality; *anneae* is in recognition of temnospondyl specialist Anne Warren.

 **Holotype.** UFPI PV001, skull and partial postcranium.

 **Paratypes.** UFPI PV004, cranium and nearly complete articulated postcranium of a probable juvenile individual; PV010 and PV281, two partial crania and pectoral girdles of probable juvenile individuals; PV351, a partial cranium of a probable juvenile.

 **Referred specimens.** UFPI PV006, PV008, PV009, PV272, PV274–PV280, PV282, PV283, PV289, PV291, PV345–PV347, PV349, PV352; various crania and postcranial elements.

 **Horizon and locality.** Lower Pedra de Fogo Formation (Parnaíba Basin), Cisuralian. The holotype, PV004, PV008 and PV010 were found within the Timon Municipality, Maranhão State; all other specimens were collected within the Nazária Municipality, Piauí State, Brazil.

 **Diagnosis.** A new member of Dvinosauria distinguished from all other dvinosaurs by the following autapomorphies: absence of prenarial snout formed by the premaxilla; combined width of both parietals less than interorbital width; maximum width of parietal subequal to that of supratemporal; occipital condyles at approximately the same level as posterior margin of skull table; presence of well-developed postglenoid area on the mandible that is much longer than glenoid fossa when seen from above. It also differs from other non-tupilakosaurid dvinosaurs by the following unique combination of characters: nares close to skull midline; well-developed rectangular to triangular tabulars; palatal and quadrate rami of pterygoid strongly differentiated from each other at the level of pterygoid corpus; foramen for internal carotid artery on the ventral surface of the parasphenoid plate is elongated and groove like; maxilla does not extend posteriorly to anterior border of the subtemporal vacuity; absence of fangs in the mandibular symphysis mesial to the marginal tooth row; presence of a parasymphyseal foramen on both sides of mandibular symphysis to accommodate the vomerine fangs.

        Trimerorhachidae Cope, 1882

       *Procuhy nazariensis* gen. et sp. nov.

            (Fig. 2a–c)

 **Etymology.** *Procuhy* from *prôt* (frog) and *cuhy* (fire) in the local Timbira language (Macro-Jê group), referring to the Pedra de Fogo Formation (‘Rock of Fire' in Portuguese, after the presence of flint); *nazariensis* refers to Nazaria Municipality, where the holotype was discovered.

 **Holotype.** UFPI PV011, partial skull and left mandibular ramus.

 **Horizon and locality.** Lower Pedra de Fogo Formation (Parnaíba Basin), Cisuralian. Nazária Municipality, Piauí State, Brazil.

**Figure 3:**
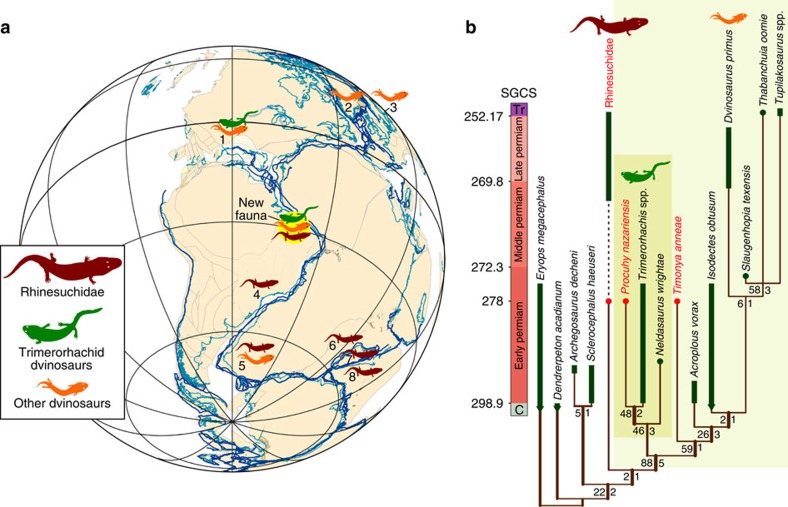
Location of the new faunal province and phylogenetic relationships of the temnospondyl taxa described herein. (**a**) reconstruction of Pangaea during the Cisuralian (278 Myr ago) showing the location of the Parnaíba Basin, and provenance and age of previous rhinesuchid and dvinosaurid temnospondyls: 1, southern USA (Cisuralian); 2, Greenland (Triassic); 3, Russian Platform (Triassic); 4, Paraná Basin (Lopingian); 5, Karoo Basin (Lopingian rhinesuchids and Triassic dvinosaurids); 6, Malawi (Lopingian); 7, Madagascar (Lopingian); 8, India (Lopingian). (**b**) Cladogram displaying the interrelationships of Rhinesuchidae with Dvinosauria and the position of *Timonya* and *Procuhy* within this group; symmetric resampling and decay index values are provided right and left of each node, respectively (symmetric resampling was calculated from 10.000 replicates [p=33] and decay index from 691 trees). Map illustrated by The PLATES Project, Institute for Geophysics, University of Texas at Austin.

 **Diagnosis.** A member of Dvinosauria distinguished from all other dvinosaurs (Fig. 3) by the following autapomorphies: length of posterior skull table greater than 90% of its width; presence of well-developed arcadian process on mandible that is longer than retroarticular process. It also differs from other trimerorhachids by the following unique combination of characters: combined width of both parietals larger than interorbital width; maximum length of the dorsal ornamented surface of tabular less than or equal to maximum length of postparietal; coronoids without denticle fields.

         Temnospondyli Zittel, 1888

         Stereospondyli Zittel, 1888

        Rhinesuchidae Watson, 1919

          gen. et sp. indet.

            (Fig. 2d–f)

 **Referred material.** UFPI PV007, partial skull table; UFPI PV003 a nearly complete right hemimandibular ramus; UFPI PV360 the posterior half of a right hemimandibular ramus.

 **Horizon and locality.** Lower Pedra de Fogo Formation (Parnaíba Basin), Cisuralian. UFPI PV007 was found at Monsenhor Gil Municipality, Piauí State, Brazil; UFPI PV003 and PV360 were collected at the Pedra de Fogo type-section in Pastos Bons, Maranhão State, Brazil.

 **Comments.** The skull material UFPI PV007 is considered to belong to a rhinesuchid stereospondyl based on the following combination of derived character states: the presence of a long and stepped jugal-prefrontal contact anterior to the orbit, anteroposteriorly elongated prefrontal located between nares and orbits, an L-shaped postorbital; orbits located posterior to mid-point of skull table; lacrimal excluded from orbit by prefrontal-jugal contact; interorbital distance less than 25% of skull width at midorbital level; jugal extends anterior to anterior orbital margin. The mandibles UFPI PV003 and PV360 are considered to belong to Rhinesuchidae based on the following synapomorphies: relatively short postglenoid area, all three coronoids covered by a field of small denticles; enlarged chordatympanic foramen located on prearticular–articular suture; prearticular with hamate process on the anterior border of glenoid fossa above the level of surangular and articular.

         Reptilia Laurenti, 1768

        Captorhinidae Case, 1911

       *Captorhinus aguti* Cope, 1882

           (Fig. 2g)

 **Referred material.** UFPI PV014, natural mould of a partial right hemimandibular ramus.

 **Horizon and locality.** Lower Pedra de Fogo Formation (Parnaíba Basin), Cisuralian. Nazária Municipality, Piauí State, Brazil.

 **Comments.** UFPI PV014 is referred to the North American *Captorhinus aguti* based on the autapomorphic presence of multiple rows of conical teeth that are aligned obliquely in relation to the long axis of the mandibular ramus^10^. UFPI PV014 can be differentiated from the small, multiple tooth-rowed captorhinid *Captorhinikos valensis*^11^, also from North America, by the following features: (a) presence of large teeth at the beginning of some tooth rows, in contrast to the distal increase of tooth size present in the tooth rows of *Captorhinikos*; (b) absence of mesial convergence of the tooth rows (tooth rows are radially oriented and distally branching in *Captorhinikos*); (c) at least one of the tooth rows is aligned along the main axis of the mandibular ramus in *Captorhinikos*, a condition not seen in UFPI PV014.

### Cladistic analysis

To elucidate the relationships of the new temnospondyls, a phylogenetic analysis was conducted, resulting in a single most parsimonious cladogram with a length of 289 steps (consistency index=0.635 and retention index=0.513; Fig. 3). The outgroups, *Eryops* and *Dendrerpeton*, fall at the base of the tree as successive sister taxa of the ingroup. The ingroup encompasses two subclades, one formed by Rhinesuchidae+Dvinosauria and the second by *Archegosaurus*+*Sclerocephalus*, the latter representing the basal radiation of stereospondylomorphs. The position of Rhinesuchidae as more closely related to Dvinosauria than to stereospondylomorphs is probably a result of the limited taxon sampling of the data set. Several more inclusive analyses have repeatedly demonstrated that rhinesuchids are stereospondyl temnospondyls^12^,^13^,^14^. Support for the most parsimonious cladogram is relatively weak, with most nodes decaying in 1–3 steps and few nodes receiving >50% symmetric resampling support.

The results of our analysis (Fig. 3) indicate that the clade Dvinosauria consists of two sister-groups, the Trimerorhachidae (consisting of *Neldasaurus*, *Procuhy* and *Trimerorhachis*) and the clade including *Timonya*, *Acroplous*, *Isodectes*, *Dvinosaurus*, and Tupilakosauridae. Therefore, both of the new taxa from Brazil are dvinosaurs, but they fall within distinct subclades. Characters that support their placement in Dvinosauria^12^ include: reduction of the otic notch; parietals more than two and a half times as long as wide; reduced anterior extension of the prefrontal, which is located around the anteromedial border of the orbit; loss of the posterolateral flange on the palatine ramus of the pterygoid; and increased number of presacral vertebrae (>30). The position of *Procuhy* within Trimerorhachidae (*Neldasaurus*+(*Trimerhorachis*+*Procuhy*)) is supported by: presence of an intertemporal on the skull table; presence of an elongated postorbital; postparietal bones that do not taper abruptly laterally; prearticular that does not extend anterior to the level of the suture of the middle and posterior coronoids; presence of a hamate process on the anterior margin of the glenoid fossa of the mandible; and, presence of a parasymphysial foramen on both sides of the mandibular symphysis. *Timonya* is reconstructed as an early-diverging dvinosaur based on the presence of postparietals that taper abruptly laterally; reduced tabulars; presence of a dorsal process of the palatine exposed on the skull roof; absence of contact between the palatine ramus of the pterygoid and the vomer; presence of a vaulted palate formed by the downturned quadrate ramus of the pterygoid; and glenoid fossa of the mandible below the level of the dorsal surface of the dentary. Although we did not recover *Timonya* as a member of Tupilakosauridae (*Slaugenhopia*+*Thabanchuia*+*Tupilakosaurus*), it is interesting to note that the Brazilian dvinosaur shares several derived character states with that clade, which we interpret as homoplasies. These are: presence of a quadrangular or rounded postorbital; anterior border of the squamosal lying approximately at the mid-length of the parietal; broad basicranial suture between the pterygoid and the parasphenoid; and posterior edge of the pterygoid incised close to the suture with the parasphenoid.

### Sedimentology and palaeoenvironment

**Figure 4:**
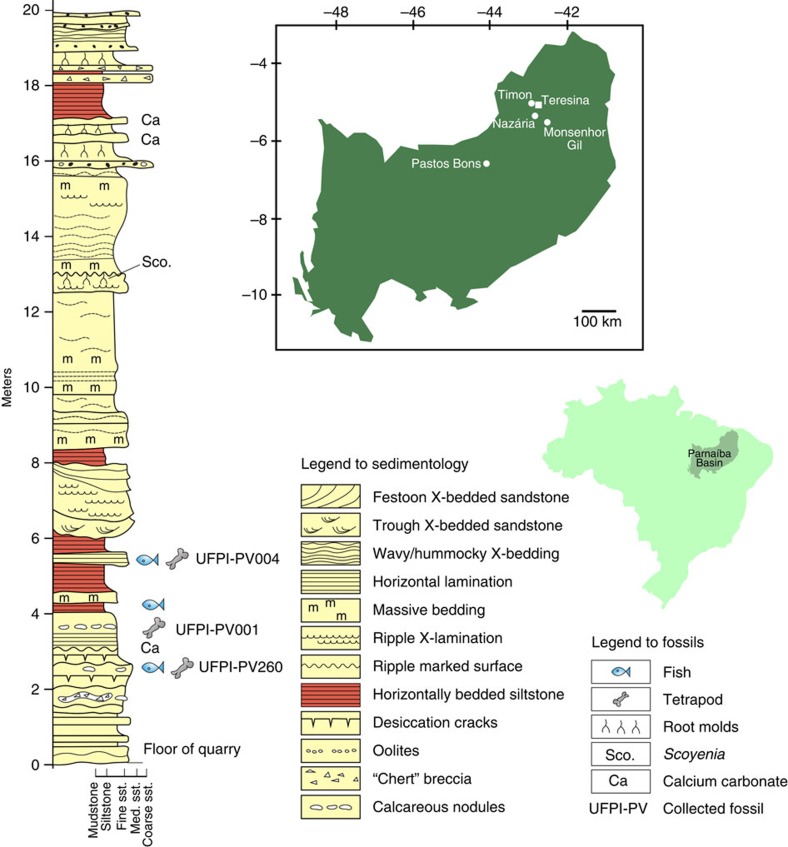
Study area in northeastern Brazil. Location of the collecting sites (represented by white dots) in the Parnaíba Basin, and sedimentological log of the Pedra de Fogo Formation, exposed in a quarry at Timon, type locality of the new dvinosaur temnospondyl *Timonya anneae*. Sst, sandstone.

Most vertebrate specimens were recovered from Pedra de Fogo outcrops near the margins of the Parnaíba Basin, in the adjacent municipalities of Nazária, Monsenhor Gil, and the capital city of Teresina, in the state of Piauí, and equivalent exposures in the municipality of Timon, in the neighbouring state of Maranhão (Fig. 4). However, previously described vertebrate fossils from the PFF, as well as some new specimens, were discovered in outcrops 220 km southwest of Teresina (Pastos Bons, Maranhão State), in strata that accumulated closer to the depocenter of the basin^5^,^15^,^16^,^17^. The presence of shared taxa in both fossil-bearing intervals suggests they represent equivalent time spans. The rocks exposed at the Timon quarry are massive, tabular strata of red siltstone interbedded with fine-grained sandstone and mudstone intervals. The fossil-bearing strata in this quarry are an ∼3 m thick succession of fissile dark reddish-brown mudstone overlying a finely laminated light-purple calcareous fine-grained sandstone with greenish grey mottles (3–6 m on Fig. 4). The wavy-laminated sandstone contains distinctive large oblate calcareous nodules and elongated chert breccia-filled tubes that resemble large rhizoliths. This sandstone grades upwards into very finely laminated dark reddish-brown mudstones with some surfaces covered in bird-foot septarian shrinkage cracks and others with oscillation ripples. The top of this mudrock unit contains lenses of fish scales and a few semi-articulated bony fishes. The sequence continues with very finely laminated dark red siltstone containing horizons of small oblate calcareous nodules that have been preferentially silicified. Fish scales occur scattered throughout this unit, and more complete remains occur around the 6 m level, where articulated temnospondyl skeletons have been recovered.

The fossil-bearing interval (3–6 m on Fig. 4) is interpreted as fluvio-lacustrine in origin, deposited in a range of terrestrial and aquatic sub-environments on the margins and nearshore of large continental lakes, on seasonally wet floodplains and channels, and within small ponds (*cf*. facies association AF2 (ref. 18)). At the 6 m mark this interval is truncated by the basal erosion surface of a large fluvial channel sandstone structured with trough cross-bedding at the base, and ripple cross-lamination in the interdigitating sandstone stringers at the top. From this level upwards the succession becomes more floodplain-dominated, with vertical rhizocretions reflecting incipient palaeosol development in a sequence of weak red-coloured massive siltstone beds (at 12 m on Fig. 4).

## Discussion

The age of the PFF/MF has been the source of continuing debate, with various authors proposing different ages based on data from plant body fossils, fossil pollen, fish remains and temnospondyl amphibians^5^,^6^,^9^,^15^,^19^,^20^. The stratigraphical range of the ctenacanth shark *Glikmanius* constrains the age between the Carboniferous and the Guadalupian^21^. Palynological studies have suggested a Cisuralian age^19^ and similarities between the pollen assemblages known from the Pedra de Fogo Formation and the Flowerpot Formation of Texas (Cisuralian–Guadalupian^22^,^23^) have been noted^19^. There is significant overlap between the fossil plant communities of the PFF/MF and the Chemnitz fossil forest of Germany, which has been dated to 278±5 Myr ago (Kungurian^6^,^9^). Furthermore, the chondrichthyan *Itapyrodus*, previously known in the PFF, was recorded in the Irati Formation^8^ of the Paraná Basin, which was dated to 278.4±5 Myr ago^9^. A Kungurian age for the PFF is further supported by our discovery of *Captorhinus aguti*, a reptile species whose last appearance is in strata attributed to the mid-Kungurian in North America^10^,^23^. It has been proposed^15^ that the amphibian *Prionosuchus* indicates a younger Guadalupian–Lopingian age, but we reject this conclusion because it is based on the apparent evolutionary grade of the taxon instead of a formal biostratigraphic correlation.

During the Permian the Parnaíba Basin was positioned in the southern tropics of western Gondwana (Fig. 3), a portion of Pangaea that so far has yielded only scarce tetrapod fossils. The nearest well-known Permian tetrapod localities in Gondwana occur further to the east in North Africa (for example, the Moradi Formation of Niger^24^) and much further south in the South American Paraná Basin^20^. However, both of these sedimentary basins have yielded younger Permian (Lopingian) fossils, which underscores the significance of this new PFF vertebrate fauna.

The PFF tetrapod assemblage includes a trimerorhachid dvinosaur, an early-diverging dvinosaur, and a captorhinid reptile. This assemblage of taxa resembles contemporaneous equatorial communities from North America that include the dvinosaurs *Trimerorhachis* and *Slaugenhopia* and several captorhinid species. It has been suggested^24^ that the unusual composition of the equatorial tetrapod assemblage of the Lopingian Moradi Formation of Niger stemmed from it being a relict of a formerly more widespread tropical biome that is best known from the Permo-Carboniferous of equatorial Euramerica. Because the PFF tetrapod assemblage is very similar to contemporaneous equatorial communities from North America^10^,^23^,^25^, it shows that the range of these equatorial communities extended into the Gondwanan tropics, providing hard evidence for the presence of an extensive Pangaean tropical biome, at least during early Permian (Cisuralian) times.

On the basis of the Texan *Slaugenhopia*^25^, tupilakosaurid dvinosaurs were hypothesized to have originated in the tropics with subsequent dispersal into higher latitudes of Pangaea, and our discovery of the new early-diverging dvinosaur *Timonya* in the PFF fauna is consistent with this claim. In addition, the presence of rhinesuchids here is unprecedented; previously this group was only known from much younger strata in southern Gondwana^26^. The new PFF rhinesuchids are the oldest known members of this clade and also represent its most northerly occurrence. Our new finds may indicate that the Cisuralian tropics were an important cradle for high-latitude biodiversity recorded later during the Permian. However, Cisuralian continental faunas are practically unknown elsewhere in Gondwana and in high-latitude Laurasia. The current absence of fossil tetrapods from these regions hampers the comparisons necessary to test whether this biogeographic pattern is real or simply an artefact of a poorly sampled fossil record. The discovery of the PFF fauna improves our understanding of early tetrapod biogeography but also raises new questions and emphasizes the necessity of further exploration of Cisuralian terrestrial biodiversity, especially in poorly studied successions deposited in the palaeotropics.

**Figure 5:**
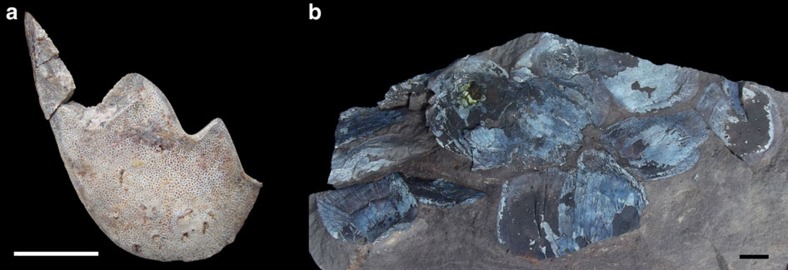
Palaeoichthyofauna from the Cisuralian of northeastern Brazil. (**a**) UFPI PV016, partial tooth-plate of a sarcopterygian fish (Dipnoi). (**b**) UFPI PV235, scales of a large sarcopterygian fish (Dipnoi). Both specimens found in Nazária, Piauí State. Scale bars represent 10 mm.

**Figure 6:**
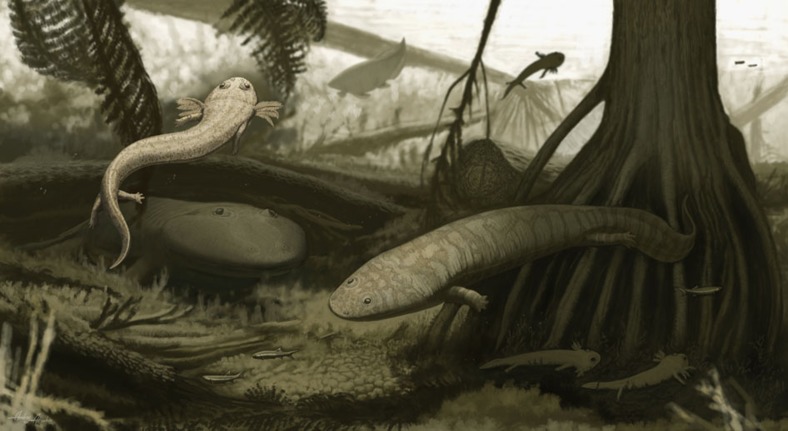
Reconstruction of the Cisuralian lacustrine/wetland community of the Teresina area in northeastern Brazil. The small dvinosaur *Timonya anneae* is depicted in the foreground left, the trimerorhachid dvinosaur *Procuhy nazariensis* is on the right, and the rhinesuchid in the left background. Other species represented are: a dipnoan, primitive actinopterygians, *Psaronius* tree-ferns, *Teresinoxylon* trees and small columnar stromatolites.

The newly discovered vertebrate assemblage from the lower PFF beds presents a compelling picture of a continental alkaline lake or wetland community and contrasts with the fauna previously reported for the parts of the basin closer to the depocenter (upper PFF beds), where the water bodies were deeper. The latter assemblage includes a greater diversity of fish and large chondrichthyans, the large piscivorous temnospondyl *Prionosuchus* and remains of small-to-medium-sized rhinesuchids. Near Teresina, undescribed primitive actinopterygian fishes and the basal dvinosaur *Timonya* represent the small faunivores; mid-level predators include the trimerorhachid *Procuhy* and large, omnivorous dipnoans (Fig. 5). The medium-sized rhinesuchid and large *Prionosuchus* occupied the highest trophic levels. Chondrichthyans are very rare in this assemblage (Fig. 6). Primary producers are well represented by stromatolites^17^, fossil trees preserved in life position^27^ and ubiquitous plant hash. Primary consumers are represented by the captorhinid and, possibly, plant-eating fish. This multi-level trophic structure parallels that recently reported for a Cisuralian freshwater community from Germany^28^, but differs in that temnospondyls occupied both the middle (dvinosaurs) and the highest (rhinesuchids and *Prionosuchus*) trophic levels.

The PFF assemblage indicates that during the Cisuralian there was an extensive Pangaean tropical/subtropical biome. It also demonstrates that life was abundant far inland during the deposition of the PFF, before more arid conditions began to prevail in the Pangaean supercontinent towards the end of the Permian.

## Methods

### Phylogenetic analysis

Several previous phylogenetic analyses and anatomical character state codings for the Dvinosauria and related groups^12^,^13^,^14^,^29^,^30^ were utilized to elucidate the relationships of the new Brazilian taxa. Thirteen temnospondyl taxa were selected (Supplementary Table 1), in addition to *Timonya* and *Procuhy* from Brazil, for analysis. Among them, *Eryops* and *Dendrerpeton* served as outgroups and *Sclerocephalus*, *Archegosaurus* and the Rhinesuchidae were chosen as representatives of the basal radiation of stereospondylomorph temnospondyls.

The character-taxon matrix was produced with Mesquite version 2.75 (ref. 31). It includes 15 temnospondyl taxa coded for 115 characters (Supplementary Note 1), 108 cranial and 7 postcranial, all considered unordered and unweighted. We analysed the final data set (Supplementary Note 2) using the Implicit Enumeration algorithm of TNT version 1.1 (ref. 32), which resulted in a single most parsimonious tree (298 steps) depicted in Fig. 3. The results are discussed further in the Supplementary Discussion.

### Computer tomography and image processing

The CT images were acquired with a Varian Medical Systems (Lincolnshire, IL, USA) model BIR-225/500-CT/DR Industrial CT system. The system uses a 10–225 kV microfocus X-ray source and an amorphous silicon flat panel detector with a pixel size of 200 microns by 200 microns and a resolution of 2,048 by 2,048 pixels. Specimen UFPI PV011 was scanned at 220 kV, 400 mA, with a 0.5 mm brass beam filter obtaining a 0.0635, mm voxel resolution. The CT data were imported into the software Amira 5.3.3, where the segmentation and the initial three-dimensional (3D) surface model were computed. The extracted 3D surface models were then imported into the Geomagic Studio 2012 software to repair and edit irregularities in the surface mesh geometry. All animations and still images of the digital specimen were rendered in the Blender 2.60 software.

### Institutional abbreviations

UFPI, Universidade Federal do Piauí, Teresina, Brazil; FMNH, Field Museum of Natural History, Chicago, USA.

### Nomenclatural acts

This published work and the nomenclatural acts it contains have been registered in ZooBank, the proposed online registration system for the International Code of Zoological Nomenclature. The ZooBank LSIDs (Life Science Identifiers) can be resolved and the associated information viewed through any standard web browser by appending the LSID to the prefix ‘http://zoobank.org/'. The LSIDs for this publication are: urn:lsid:zoobank.org:pub:5A7C9C3D-F7B1-43FF-896A-67D0BC3B00C6

## Additional information

**How to cite this article:** Cisneros, J. C. *et al*. New Permian fauna from tropical Gondwana. *Nat. Commun.* 6:8676 doi: 10.1038/ncomms9676 (2015).

## Supplementary Material

Supplementary Information Supplementary Figure 1, Supplementary Table 1, Supplementary Note 1-2, Supplementary Discussion and Supplementary References

Supplementary Movie 1 CT-rendering of specimen UFPI PV001, holotype of Timonya anneae, cranium and partial postcranium. Rotation around the long axis of the skeleton.

## Figures and Tables

**Figure 2:**
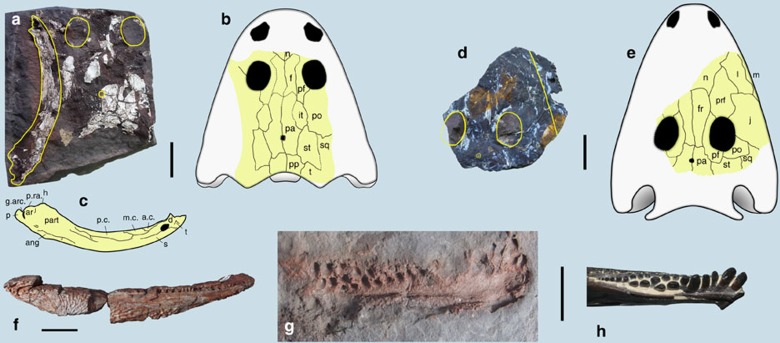
Tetrapods from the Cisuralian of northeastern Brazil. (**a**–**c**) The trimerorhachid *Procuhy nazariensis* n. g. n. sp. (UFPI PV011), skull roof in ventral view and left hemimandibular ramus partially preserved in lingual view. a.c., anterior coronoid; ang, angular; ar, articular; d, dentary; f, frontal; fr, frontal; g.arc, arcadian groove; h, hamate process; it, intertemporal; j, jugal; l, lacrimal; m.c., middle coronoid; m, maxilla; n, nasal; p, parietal; pa, parietal; part, prearticular; p.c., posterior coronoid; pf, postfrontal; po, postorbital; pp, postparietal; prf, prefrontal; p.ra, retroarticular process; s, splenial; sq, squamosal; st, supratemporal; t, tabular; t, tooth. (**d**,**e**) partial skull table of a rhinesuchid (UFPI PV007) in ventral view, (**f**), right hemimandibular ramus of a rhinesuchid (UFPI PV003). f, frontal; fr, frontal; it, intertemporal; j, jugal; l, lacrimal; m, maxilla; n, nasal; p, parietal; pa, parietal; pf, postfrontal; po, postorbital; pp, postparietal; prf, prefrontal; sq, squamosal; st, supratemporal; t, tabular. (**g**) Right hemimandibular ramus of Captorhinus aguti, natural mould (UFPI PV014), anterior to the right. (**h**) Left hemimandibular ramus of *Captorhinus aguti*, in occlusal view, from Oklahoma, USA (FMNH PR 2107). Scale bar, 50 mm (**a**–**e**) 200 mm (**f**) and 10 mm (**g**,**h**).
