# Supplementary material for: New Permian fauna from tropical Gondwana
Source: Nat Commun. 2015 Nov 5;6:8676. doi: 10.1038/ncomms9676 (PMC4659833; doi:10.1038/ncomms9676)
Supplement: Supplementary Information — Supplementary Figure 1, Supplementary Table 1, Supplementary Note 1-2, Supplementary Discussion and Supplementary References [file ncomms9676-s1.pdf]

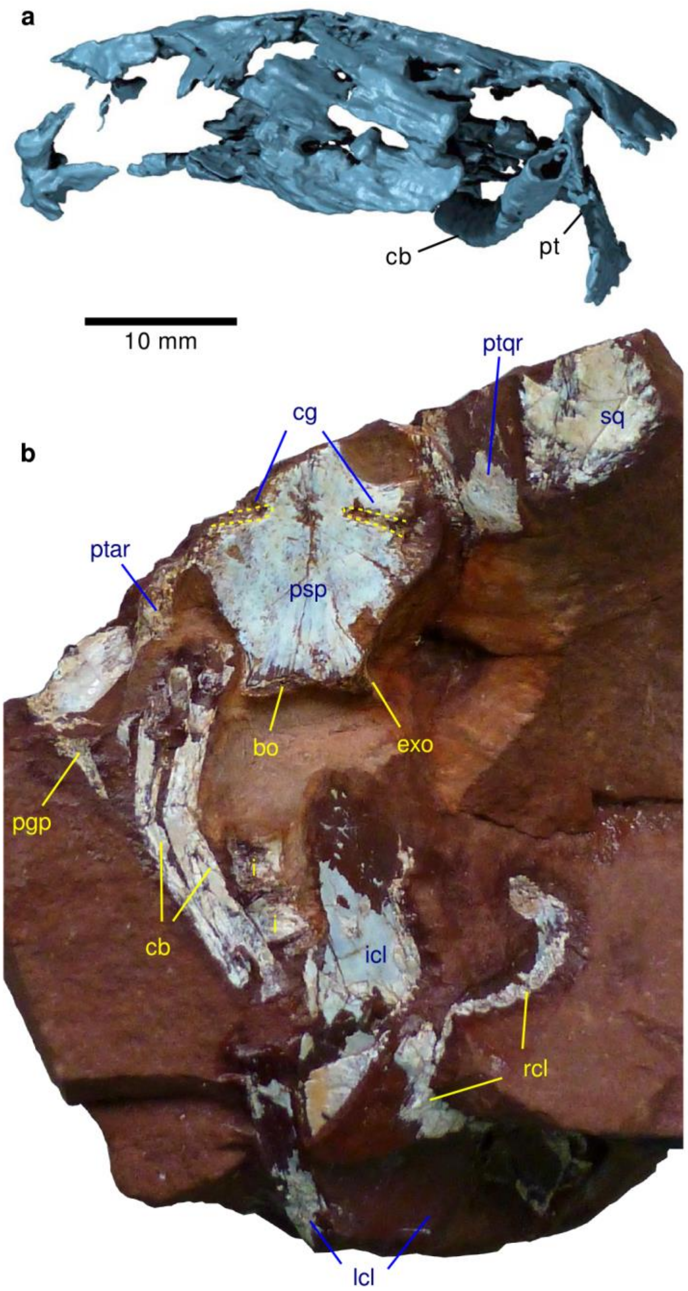

Supplementary Figure 1 | Additional diagnostic information on the dvinosaur temnospondyl *Timonya anneae* gen. et sp. nov. from the lower Permian of northeastern Brazil. (**a**) Occipital view of *Timonya anneae*, holotype UFPI PV001, showing the vaulted palate. (**b**) Ventral view of *Timonya anneae*, holotype UFPI PV001, partial basicranium, hyoid apparatus, and pectoral elements, showing characters of diagnostic significance. Abbreviations: bo, basioccipital; cb, ceratobranchial; cg, carotid groove; exo, exoccipital; i, intercentrum; icl, interclavicle; lcl, left clavicle; psp, parasphenoid; pt, pterygoid; ptar, pterygoid ascending ramus of the jaw; ptqr, pterygoid quadrate ramus; pgp, postglenoid process of the jaw; rcl, right clavicle; sq, squamosal.

Acroplous vorax	USNM 22528; ([1])
Archegosaurus decheni	MCZ*; ([2])
Dendrerpeton acadianum	([3])
Dvinosaurus primus	([4]), ([5]), ([6])
Eryops megacephalus	USNM*; ([7]), ([8])
Isodectes punctulatus	([9])
Neldasaurus wrightae	MCZ 2200; ([10])
Rhinesuchidae	BPI*, SAM*
Sclerocephalus hauseri	([11])
Slaugenhopia texensis	FMNH UR155, UR702; ([12])
Tabanchuia oomie	([13])
Trimerorhachis insignis	MCZ 1508, FMNH UC646; ([14])
Tupilakosaurus sp.	([15]), ([16]), ([17])

Supplementary Table 1 | Specimens examined for character codings. List of the specimens personally examined by one or more authors and the references utilised in the present study. An asterisk following the institution acronyms indicates that several specimens of the taxon were examined in the corresponding institution. **USNM**, National Museum of Natural History, Washington, DC, USA; **MCZ**, Museum of Comparative Zoology, Harvard University, Cambridge, USA; **BPI**, Evolutionary Studies Institute of the University of the Witwatersrand, Johannesburg, South Africa; **SAM**, Iziko South African Museum, Cape Town, South Africa; **FMNH**, Field Museum of Natural History, Chicago, USA.

Supplementary Note 1. Character list

The following list of characters was utilised in the phylogenetic analysis. The characters are in part based on personal observations of the original material and others are adapted from previous analyses [1,4,18,20–22,]. All characters were treated as unordered unless indicated.

Skull table.

1. Orbits located about halfway along the skull table length [0]; orbits located in front of the mid-length of the skull [1]; orbits located behind mid-length of the skull [2].
2. Lateral border of the orbits formed only by the lacrimal and jugal (0); lateral edge of the corpus of the palatine exposed dorsally in the lateral margin of the orbit (LEP "lateral exposure of the palatine"[23]) [1]; lacrimal excluded from orbit by prefrontal-jugal contact [2].
3. Interorbital distance less than 25% of the width of the skull at midorbital level [0]; interorbital distance greater than 25% of the width of the skull at midorbital level [1].
4. Skull margins straight or slightly concave when see from above [0]; slightly convex [1].
5. Contour of the cheek in occipital view: slightly convex [0]; straight [1].
6. Length of the posterior skull table between 70% and 90% of its width (length measured as the sagittal distance from the level of the posterior edge of the orbits to the posterior margin of the skull roof; width measured as the width from the lateral edge of one tabular to the other) [0]; length of the posterior skull table 50–65% of the width [1]; length of posterior skull table less than 46% of the width [2]; length of posterior skull table greater than 90% of the width [3].
7. Sculpture of the dorsal skull roof forms a 'normal' ridge-grooved pattern without pustules or nodules on the junctions [0]; spider-web pattern with pustules or nodules on the junctions of crests and ridges [1]; only pustules are present [2].
8. Nares not close to skull midline (distance between nares twice width of one naris or greater) [0]; nares close to skull midline (distance between nares approximates width of one naris) [1].
9. Nares rounded [0]; narrow and anteroposteriorly elongated [1]; pear-shaped so they narrow anteriorly [2].
10. Prenarial snout formed by an anterior expansion of the premaxilla present [0]; absent [1].
11. Prenarial skull length less than internarial distance [0]; prenarial skull length equal to or up to three times the internarial distance [1]; length of the snout greater than three times the internarial distance [2].
12. Snout margins continually converging towards the tip [0]; tip of snout expanded so that the snout margins are parallel, or snout margins concave before the tip [1].
13. Sensory sulci well developed [0]; present but poorly developed [1]; deeply incised between orbit and nostril only [2]; sensory sulci absent [3].
14. Infraorbital sulcus straight or gently curved [0]; infra-orbital sulcus with a step or S-like flexure between the orbit and the naris [1]; infra-orbital sulcus ‘Z’ shaped with a very tight flexure [2].
15. Lacrimal bones present [0]; absent [1].
16. Premaxilla with a triangular posterior process medial to the naris [0]; triangular process absent and the posterior dorsal margin of the premaxilla forms a simple suture with the nasal [1].
17. Contact between premaxilla and nasal anterior to the nares [0]; at level of the nares [1]; posterior to the nares [2].
18. Septomaxilla part of the skull roof forming the posterior nasal margin [0]; septomaxilla part of the skull roof and with a well developed intranarial process [1]; septomaxilla restricted to the floor of the nasal cavity with limited or no contact with the posterior nasal margin [2]; unossified [3].
19. Jugal extends anterior to anterior orbital margin [0]; anterior end of jugal about level with or posterior to anterior orbital margin [1].

20. Prefrontal and jugal not in contact [0]; prefrontal and jugal form a nearly straight suture anterior to the orbit [1]; long and stepped jugal-prefrontal contact anterior to the orbit [2].
21. Prefrontal does not contribute to the margin of the external naris [0]; prefrontal contributes to the margin of the external naris [1].
22. Prefrontal crescent-shaped and curves around the anteromedial border of the orbit [0]; anteroposteriorly elongated prefrontal located between the nares and orbits [1].
23. Prefrontal-maxilla contact absent [0]; present [1].
24. Prefrontals and postfrontals form a suture [0]; prefrontals and postfrontals fail to contact so that the frontal contributes to the orbital margin [1].
25. Prefrontal and postfrontal suture anterior to the midpoint of the orbit [0]; situated approximately at or posterior to the midpoint of the orbit [1].
26. Postorbital antero-posteriorly elongated [0]; postorbital quadrangular or rounded but not anteroposteriorly elongated [1]; postorbital L-shaped ("hooked" *sensu* Damiani 2001 [[24]]) [2].
27. Postfrontal not reduced [0]; very reduced and allowing a postorbital-frontal contact [1].
28. Intertemporal: present [0]; absent [1].
29. Maxilla and nasal not in contact [0]; maxilla and nasal form a suture [1].
30. Maxilla and quadratojugal form a suture or a point contact on the ventral border of the cheek [0]; maxilla and quadratojugal do not contact [1].
31. Maxilla extends posterior to the anterior border of the subtemporal vacuity [0]; maxilla does not extend posterior to the anterior border of the subtemporal vacuity [1].
32. Anteroposterior length of the supratemporal shorter than the length of the squamosal [0]; as long as the squamosal [1].
33. Parietal bones project posterolaterally (L-shaped) [0]; parietals quadrangular [1].
34. Anteriormost extension of the external surface of the squamosal lying posterior to the parietal midlength [0]; in front [1]; approximately level with the parietal midlength [2].
35. Parietal-postorbital contact: absent [0]; present [1].
36. Combined transverse width of the postparietals less than four times the anteroposterior length of the postparietal [0]; combined transverse width greater than four times the length [1].
37. Length of each parietal more than two and a half times its width [0]; parietal less than two and a half times long as wide [1].
38. Combined width of both parietals approximately equal to the interorbital width [0]; smaller than the interorbital width [1]; larger than interorbital width [2].
39. Maximum width of the parietal greater than that of the supratemporal [0]; subequal to that of the supratemporal [1]; less than that of the supratemporal [2].
40. Postparietals do not taper abruptly laterally [0]; tapering [1].
41. Postparietals do not form an L-shaped ("stepped") suture with the supratemporals [0]; postparietals do form an L-shaped ("stepped") suture with the supratemporals [1].
42. Tabulars are well developed rectangular to triangular bones [0]; tabulars reduced to thin slivers lying against the posterior margin of the skull roof [1].
43. Tabular horns end at the same level as or anterior to the posterolateral corners of the skull roof [0]; tabular horns extend posterior to the posterolateral corners of the skull roof [1].
44. Maximum length of the dorsal ornamented surface of the tabular greater than one-third of the maximum length of the postparietal [0]; less than or equal the maximum length of the postparietal [1].
45. Otic notch deep [0]; otic notch is a wide shallow embayment [1]; otic notch absent [2].
46. Squamosal and tabular separated by the supratemporal [0]; squamosal-tabular suture present on the dorsal skull roof [1].

Occiput.

47. Occipital condyles do not project beyond the posterior margin of the skull table [0]; condyles project beyond the posterior margin of the skull table [1]; condyles at approximately the same level of the posterior margin of the skull table [2].

48. Quadrate condyles well posterior to the occipital condyles [0]; in approximately the same transverse plane as the occipital condyles [1]; anterior to the occipital condyles [2].
49. Ascending ramus of the pterygoid contacts the squamosal [0]; ascending ramus does not contact the squamosal creating an upper palatoquadrate fissure [1].
50. Ascending ramus of the pterygoid thickened by an ascending column positioned towards its medial edge [1]; column absent [0].
51. Ascending ramus of the pterygoid forms a continuous curve with the posterior edge of the quadrate ramus [0]; ascending ramus of the pterygoid arises from the dorsal surface of the pterygoid as a shallow curved lamina [1]; ascending ramus of the pterygoid arises from the dorsal surface as a shallow uncurved lamina [2]; ascending ramus of the pterygoid arises from the dorsal surface as a gently concave lamina which is also curved posteriorly in vertical section [3]; ascending ramus of the pterygoid arises from the dorsal surface as a gently concave lamina not curve posteriorly [4].
52. Posterior face of quadrate ramus of the pterygoid lacking an oblique ridge [0]; a large, sharp lamina (oblique ridge) rises vertically from the occipital surface of the quadrate ramus at the level of the dorsalmost tip of the quadrate when see from behind and partially conceals the ascending ramus in occipital view [1]; the oblique ridge is a relatively low lamina that does not conceal the ascending ramus from behind [2].
53. Post temporal fenestra markedly wider than deep [0]; about as wide as deep or deeper than wide [1]; post temporal fenestra reduced to small foramen or entirely closed [2]; post temporal fenestra slit-shaped [3].
54. Quadratojugal foramen absent [0]; present on the occipital surface of the quadratojugal but occupying less than one third of the width of the surface [1]; present and occupying at least one-third of the surface width [2].
55. Quadrate ramus of the pterygoid level with palate [0]; quadrate ramus slightly downturned [1]; quadrate ramus strongly downturned [2].

Palate.

56. Ceratobranchials absent or unossified in adults [0]; present in adults [1].
57. Anterior palatal fossa not perforated [0]; anterior palatal fossa perforated to form an anterior palatal vacuity [1].
58. Maxilla and vomer not in contact or in point contact [0]; maxilla and vomer forming a suture [1].
59. Vomer contacts the palatine ramus of the pterygoid on the anterior edge of the interpterygoid vacuity [0]; palatine ramus of the pterygoid contacts the vomer on the anterolateral border of the interpterygoid vacuity [1]; palatine ramus of the pterygoid not in contact with the vomer [2].
60. Palatine ramus of the pterygoid meets palatine on the lateral margin of interpterygoid vacuity [0]; pterygoid retracted so ectopterygoid is exposed in the interpterygoid vacuity and contributes to strut between interpterygoid and subtemporal vacuities [1]; pterygoid markedly retracted so ectopterygoid makes a large contribution to strut between interpterygoid and subtemporal vacuities [2].
61. Palatine ramus of the pterygoid bears a posterolateral flange [0]; flange absent [1].
62. Posterior edge of the ectopterygoid contributes to the anterior margin of the subtemporal fossa [0]; posterior end of the ectopterygoid separated from the subtemporal fossa by an alar process of the jugal [1].
63. Width of interpterygoid vacuity pair less than 90% of their length [0]; width of pair greater than 90% of their length [1].
64. Maximum width of the interpterygoid vacuities anterior to the midpoint of the vacuity [0]; approximately at mid-length of the vacuity [1]; posterior to the midpoint of the vacuity [2].
65. Parasphenoid articulates with depressions in the corpus of the pterygoid [0]; an elongate cylindrical to hemicylindrical medial process of the pterygoid abuts the parasphenoid [1];

corpus of the pterygoid forming a flat suture (basicranial suture) with the parasphenoid plate [2]; the basicranial suture extends anteroposteriorly along the lateral margins of the parasphenoid plate for more than the 50 % of the total length of the plate [3].

66. Foramen for the internal carotid artery on the ventral surface of the parasphenoid plate [0]; foramen for the internal carotid located on the lateral side of the parasphenoid plate, posterior to the pterygoid articulation [1]; internal carotid and the palatine and intracranial branches pass through the dorsal surface of the parasphenoid plate, either leaving foramina where they pass below the surface or grooves where they lie upon it [2]; internal carotid artery passes along the ventral surface of the parasphenoid plate, leaving a groove, with a foramen on the lateral side of the parasphenoid at the base of the cultriform process, where the intracranial branch enters the parasphenoid [3].
67. The width of the cultriform process of the parasphenoid at its midpoint is less than 10% of the length of the process (length of the cultriform process measured as the length between the anterior and posterior ends of the interpterygoid vacuities) [0]; width of cultriform process more than 10% of its length [1].
68. Ventral surface of the cultriform process narrow and rounded [0]; gently convex [1]; with flat ventral surface and unexpanded anteriorly [2]; flat and expanded anteriorly between the vomers [3]; ventral surface of the cultriform process flat with a median keel [4].
69. Cultriform process of the parasphenoid projects anteriorly between the vomers beyond the anterior border of the interpterygoid vacuities [0]; cultriform process ends at the level of or behind the anterior border of the interpterygoid vacuities [1].
70. Vomerine depression or foramen just anterior to cultriform process of the parasphenoid absent [0]; present [1]; present as a vacuity [2].
71. Prefenestral division of the palate (vomerine plate anterior to the interpterygoid vacuities) wider than long [0]; longer than wide [1]; approximately as wide as long [2].
72. Posterior extension of the vomer present on the lateral border of the interpterygoid vacuity until the level of the palatine fangs [0]; posterior extension of the vomer absent [1].
73. Ectopterygoid with enlarged tusks at its anterior end [0]; ectopterygoid tusks absent [1].
74. Ectopterygoid with only one or two teeth [0]; ectopterygoid with a tooth row of more than three teeth [1]; ectopterygoid with two rows of teeth [2]; ectopterygoid without teeth [3].
75. Absence of a tooth row behind the palatine tusks [0]; four to six palatine teeth [1]; more than seven palatine teeth [2].
76. Medial margin of the choana without teeth [0]; medial margin of the choana with a row of teeth [1].
77. Vomers without a row of teeth between the vomerine fangs [0]; with a straight tooth row running transversely between the vomerine fangs [1]; transverse vomerine tooth row ‘V’ shaped [2]; transverse vomerine tooth row anteriorly concave [3]; transverse vomerine tooth row anteriorly convex [4].
78. Vomerine fangs subparallel to the marginal tooth row [0]; not subparallel [1].
79. Palatal and quadrate ramus of the pterygoid well differentiated from one another at the level of the pterygoid corpus [0]; palatal and quadrate ramus of the pterygoid poorly differentiated and forming a continuous sheet of bone on the medial border of the subtemporal vacuity [1].
80. Posterior edge of the pterygoid incised close to the suture with the parasphenoid, [1]; not incised [0].
81. Basioccipital-pterygoid contact absent [0]; present [1].
82. Elongate grooves flanked by distinct ridges that run anteroposteriorly to posterolaterally on the ventral surface of the parasphenoid plate from a point situated behind the basipterygoid processes to a posterolateral notch visible along the lateral margin of the plate: absent [0]; present [1].
83. Crista muscularis of parasphenoid present at the level of posterior border of pterygoid-parasphenoid suture [0]; present posterior to the posterior border of pterygoid-parasphenoid suture [1]; crista muscularis absent [2].

84. "Pockets" situated on ventral surface of the corpus of the parasphenoid [0]; excavated on the corpus of the parasphenoid so that they are situated dorsally to the ventral surface of the bone [1].
85. Parasphenoid plate subrectangular and longer than wide [0]; parasphenoid plate slightly wider than long or as wide as long [1]; parasphenoid plate expanded transversely creating lateral ‘wings’ [2].
86. Ventral surface of the pterygoids without ornament [0]; with ornament [1].
87. Ventral surface of the parasphenoid corpus without ornament [0]; with ornament [1].
88. Pterygoids without denticles [0]; pterygoids covered by denticles [1].
89. Teeth more or less rounded in cross section close to their base [0]; teeth compressed and labiolingually elongated in cross section [1].

Mandible.

90. No extension of the mandible behind the glenoid fossa [0]; a relatively short postglenoid area (PGA[25]) present behind the glenoid fossa on the mandible [1]; a well developed postgenoid area present, much longer than the glenoid fossa when seen from above [2].
91. No arcadian groove[25] at the posterior end of the surangular in the PGA [0]; arcadian groove present [1].
92. Prearticular extends anteriorly to the level of the midpoint of the middle coronoid [0]; prearticular does not extend anterior to the level of the suture of the middle and posterior coronoids [1]; prearticular extends anteriorly to reach the symphysis [2].
93. Anterior Meckelian foramen absent [0]; single anterior Meckelian foramen [1]; two or more anterior Meckelian foramina [2].
94. Posterior Meckelian foramen bordered by the prearticular, postsplenial and the angular [0]; posterior Meckelian foramen bordered by the prearticular and postsplenial exclusively [1]; posterior Meckelian foramen bordered by the prearticular and angular [2].
95. Length of the posterior Meckelian foramen greater than 50% of the length of the adductor fossa [0]; length of the posterior Meckelian foramen less than a 50% of the length of the adductor fossa [1].
96. Parasymphysial foramen on both sides of the symphysis absent [0]; parasymphysial foramen present [1].
97. All three coronoids bear a field of small denticles [0]; field of denticles restricted to the posterior coronoid [1]; coronoids without any denticle fields [2].
98. Mandibular sulcus shallow at its posterior end [0]; mandibular sulcus deeply incised at its posterior end [1].
99. Postglenoid process on the posterior end of the mandible no longer than the arcadian process [0]; postglenoid process much longer than the arcadian process [1]; postglenoid process absent [2].
100. Chorda tympanic foramen located on the suture between the articular and the prearticular [0]; chorda tympanic foramen located on the prearticular alone [1]; chorda tympanic foramen absent [2].
101. Chorda tympanic foramen large as the anterior Meckelian foramen [0]; chorda tympanic foramen smaller than the anterior Meckelian foramen [1].
102. Prearticular without a hamate process [0]; hamate process present on the anterior margin of the glenoid fossa above the level of the dorsal surfaces of the surangular and articular [24] [1].
103. Coronoid bones without teeth other than denticles [0]; posterior coronoid with a row of teeth [1]; all coronoids with a continuous tooth row [2]; fangs on the coronoids [3].
104. Transverse trough on the dorsal surface of the retroarticular process: absent [0]; present [1].
105. Ventral margin of angular smoothly curved [0]; nearly flat for most of its length [1].
106. Posterior coronoid not visible in labial view [0]; visible in labial view [1].
107. Labial wall of adductor fossa nearly horizontal [0]; strongly convex dorsally [1].
108. Glenoid fossa above level of dorsal surface of dentary [0]; below level of dorsal surface of

dentary [1].

Postcranium.

109. Posterior margin of the interclavicle does not form a parasternal process [0]; forms a parasternal process [1].
110. Length of the part of the humerus shaft that lies proximal to the entepicondyle smaller than the maximum width of the humerus head [0]; greater [1].
111. Iliac blade with nearly parallel margins [0]; iliac blade flared proximally [1].
112. Iliac blade strongly posteriorly inclined [0]; nearly vertical [1].
113. Ventral edges of adjacent intercentra in contact [0]; prevented from contact by intervening pleurocentra [1].
114. Presacral intercentra open dorsally (crescentic) [0]; at least some intercentra form a complete disc [1].
115. Presacral count: less than 30 vertebrae [0]; more than 30 vertebrae [1].

Supplementary Note 2. Character states

Eryops megacephalus
2211110000 113-001001 0100010101 0011001110 0010012000 000010000- 0002111010 1000000110 0010100100 -022100021 1000011000 01100

Sclerocephalus hauseri
0200100010 011-001001 0100000110 0011021021 100000000? 001?11000- 0002010010 0111100010 002-111100 -2121?0?22 -00001100? 01100

Archegosaurus decheni
2210100010 1110001001 0100000110 0010000020 0000000000 000000000-0002130000 1011110010 0010000100 -01?100122 -000010000 11100

Benthosuchus sushkini
2210010010 1102011301 0100010111 0001000020 0010012000 0201101120 1101320010 1011112010 0000110112 1100101100 111000000? ??100

Rhinesuchidae
2200110021 -010011102 0100010111 0001011121 1000002000 013?00011- 0101320011 0001114110 0011110111 1021001120 01000100?? ???0?

Dendrerpeton acadianum
0010100000 013-000010 0100100000 0011001210 000000210? ???0000?0-1002010010 1100000110 001-000100 -?2010?000 1??-0??000 00100

Trimerorhachis insignis
1010100000 0011000010 0000000000 0100000220 000011010? 020211101-1002010000 0111111010 010-010101 1110111101 1110000001 10101

Neldasaurus wrightae
0010000110 0000010310 0000100011 0011000220 01-021000? 321?0?111-10020?1300 1001100010 012-1???01 111010?10? 1??000?001 1110?

Perryella olsoni
01110011?1 003-0???10 0001-00100 0011010221 11-100211? 32321?0120 0?11020210 0100000010 012-000101 11?210??01 1??00???00 ??10?

Isodectes punctulatus
1111000000 001?001210 1[01]00000001 1111000021 11-121121? ????20?020 11000?00?0 0?????0010 002-1???01 ?????????? ???111111? 0????

Acroplous vorax
1111011000 000?011310 0000000100 0111010021 11-1201200 30022?1120 1000011400 0103000000 002-01010[12] [01]01??011?? ?000110100 0100?

Dvinosaurus primus
0111110000 0000010310 0000010100 0111111221 01-121111? 301221111-1111011300 0101013100 002-000?01 0000102121 1021111110 00101

Slaugenhopia texensis
1?1??300?? ??0??01??? ?0?0??1??? ??1?10??21 1??1?????? ??????????
????3013?? ?????????1 ?02-0?0??? ?????????? ????1????? ?????

Tupilakosaurus sp.
111100010? ?0010???10 0010111111 0112100021 11-1211100 303221?121 101031130?
2101100101 102-0????1 0??????12? ???11111?? ???1?

Thabanchuia oomie
111100000? ?001???310 10101111?? 0112100020 01-0211210 303?21?121 11103?1201
1103010001 102-0000?1 00???02122 -03101101? ??011

Timonya anneae
1110000101 00??0??310 0000010100 1112000111 10-0212000 30?221?120 0111301?10
0?0111?011 002-100?02 ??1?112?21 100?011100 ??101

Procuhy nazariensis
1?1??30??? ??0?????10 ???00000?? ??0?000020 00?1?????? ??????????
?????????? ?????????? ?????????1 11???12??? ?1000??0?? ?????

Supplementary Discussion

The composition of Dvinosauria obtained through the present analysis is very similar to that presented in previous cladistic studies[1,4,18–20], particularly that of Ruta and Bolt[18]. Those analyses differ from each other mainly in the number of characters and taxon sampling. Including non-stereopondyl temnospondyls in particular appears to affect the resulting topologies. Yates and Warren's analysis[4] produced a Dvinosauria clade that included Trimerorhachidae (*Trimerorhachis* + *Neldasaurus*) as the sister-group of a clade containing Dvinosauroidea. The latter is therein composed of the Eobrachyopidae (*Isodectes* + *Acroplous*) and a clade that includes *Dvinosaurus* + Tupilakosauridae (*Tupilakosaurus* + 'undescribed taxon' which was later described as *Thabanchuia oomie*).

Ruta and Bolt's analysis[18] resulted in a monophyletic Dvinosauria with the Carboniferous *Eugyrinus* as the most basal taxon and Trimerorhachidae and the newly redescribed taxon *Perryella olsoni* as successive sister taxa to Dvinosauroidea. Their topology for Dvinosauroidea was equivalent to that of Yates and Warren[4], with the Eobrachyopidae (*Isodectes* + *Acroplous*) as the sister-group of *Dvinosaurus* + Tupilakosauridae (*Slaugenhopia* + (*Tupilakosaurus* + *Thabanchuia*)). Subsequently, in their redescription of *Acroplous vorax*, Engelhorn *et al.*[1] performed a new analysis in order to assess the relationships of Eobrachyopidae, albeit with the smallest number of sampled taxa among the studies considered here. Their resulting topology features a Dvinosauria that is essentially equivalent to that of Ruta and Bolt[18], with a monophyletic Trimerorhachidae and *Perryella*, *Isodectes, Acroplous*, and *Dvinosaurus* falling as successive sister taxa of Tupilakosauridae (an unresolved node uniting *Slaugenhopia* + *Tupilakosaurus* + *Thabanchuia*). Englehorn *et al.*[1] did not recover a monophyletic Eobrachyopidae and they did not include *Eugyrinus* in the analysis. Finally, Schoch's analysis[19] of Temnospondyli was based on an extensive dataset in terms of both taxon and character sampling, although he excluded *Slaugenhopia* and *Perryella*. His analysis[19] recovered a monophyletic Dvinosauria, but *Dvinosaurus* appeared in a more basal position compared to previous analyses and Eobrachyopidae and Tupilakosauridae resolved as sister groups. He also failed to recover a monophyeltic Trimerorhachide.

Our dataset is mainly focused on Dvinosauria and includes fewer taxa than those in the previous analyses[1,4,18–20], but our results are essentially equivalent. Dvinosauria is recovered as a well supported (Decay index 5, see Fig. 3) monophyletic group, which includes two subclades: Trimerorhachidae plus Tupilakosauridae and its allies. The main difference between our analysis and those of other authors is the content and order of the taxa on the tupilakosaurid stem. In order to check the stability of the Brazilian taxa as representatives of the two main dvinosaur clades we ran

our matrix with *Perryella*[18] included. Although previously considered a dvinosaur, it has recently been suggested that it is more probably a dissorophoid[20]. The inclusion of *Perryella* in our analysis did not change the position of *Procuhy* as the sister-taxon of *Trimerorhachis* or the position of *Timonya* at the base of the clade containing Tupilakosauridae and allied taxa. *Perryella* nested within the non-tupilakosaurid dvinosaurs and slightly altered the arrangement of taxa above *Timonya*: (*Timonya* + (*Perryella* + (*Dvinosaurus* + (*Acroplous* + (*Isodectes* + (Tupilakosauridae))))).

Supplementary References

1. Englehorn, J. Small, B. J. & Huttenlocker, A. A redescription of *Acroplous Vorax* (Temnospondyli: Dvinosauria) based on new specimens from the Early Permian of Nebraska and Kansas, U.S.A. *J. Vert. Paleontol.* **28,** 291–305 (2008).
2. Witzmann, F. Cranial morphology and ontogeny of the Permo-Carboniferous temnospondyl *Archegosaurus decheni* Goldfuss, 1847 from the Saar–Nahe Basin, Germany. *Trans. R. Soc. Edinb. Earth Sci.,* **96,** 131–162 (2006).
3. Holmes, R. B., Carroll, R. L. & Reisz, R. R. The first articulated skeleton of *Dendrerpeton acadianum* (Temnospondyli, Dendrerpetontidae) from the Lower Pennsylvanian locality of Joggins, Nova Scotia, and a review of its relationships. *J. Vertebr. Paleontology* **18,** 64–79 (1998).
4. Yates, A. M. & Warren, A. The phylogeny of the 'higher' temnospondyls (Vertebrata: Choanata) and its implications for the monophyly and origins of the Stereospondyli. *Zool. J. Linn. Soc.* **128,** 77–121 (2000).
5. Bystrow, A. P. *Dvinosaurus* als neotenische Form der Stegocephalen. *Acta Zool.* 19, 209-295 (1938).
6. Shishkin, M. A. The morphology of the early Amphibia and some problems of the lower tetrapod evolution. *Trudy Paleontologicheskogo Instituta* **137,** 1–260 (1973).
7. Sawin, H. J. *The cranial anatomy of* Eryops megacephalus. Thesis, Museum of Comparative Zoology (1941).
8. Pawley, K. A. T., & Anne Warren. The appendicular skeleton of *Eryops megacephalus* Cope, 1877 (Temnospondyli: Eryopoidea) from the Lower Permian of North America. *J. Paleontol.* **80,** 561–580 (2006).
9. Sequeira, S. E. K. The cranial morphology and taxonomy of the saurerpetontid *Isodectes obtusus* comb. nov. (Amphibia: Temnospondyli) from the Lower Permian of Texas. *Zool. J. Linn. Soc.* **122,** 237–259 (1998).
10. Chase, J. N. *Neldasaurus wrightae*: A New Rhachitomous Labyrinthodont from the Texas Lower Permian. *Bull. Mus. Comp. Zool.* **133,** 153–225 (1965).
11. Schoch R. R. & Witzmann F. Osteology and relationships of the temnospondyl genus *Sclerocephalus*. *Zool. J. Linn. Soc.* **157,** 135–168 (2009).
12. Milner, A. R. & Sequeira, S. E. K. *Slaugenhopia texensis* (amphibia: temnospondyli) from the Permian of Texas is a primitive tupilakosaurid. *J. Vertebr. Paleontology* **24,** 320–325 (2004).
13. Warren, A. A. 1999. Karroo tupilakosaurid: a relict from Gondwana.*Transactions of the Royal Society of Edinburgh: Earth Sciences* 89:145–160
14. . Milner, A. R. & Schoch, R. R. N. *Trimerorhachis* (Amphibia: Temnospondyli) from the Lower Permian of Texas and New Mexico: cranial osteology, taxonomy and biostratigraphy. *Jb. Geol. Paläont. Abh.* **270,** 91–128 (2013).
15. Nielsen, E. *Tupilakosaurus heilmani* Nielsen n. g. et n. sp., an interesting Batrachomorph from the Triassic of East Greenland. *Meddelelser om Granland* **72,** 1–33 (1954).
16. Nielsen, E. New observations on the skull roof of the holotype of *Tupilakosaurus heilmani* Nielsen. *Zool. J. Linn. Soc.* **47,** 225–229 (1967).
17. Shishkin, M. A. New data on *Tupilakosaurus*. *Dokl. Akad. Nauk.* **136,** 938–941 (1961).
18. Ruta, M. & Bolt, J. R. A reassessment of the temnospondyl amphibian *Perryella olsoni* from the Lower Permian of Oklahoma. *Trans. R. Soc. Edinb. Earth Sci.* **97,** 113–165 (2006).
19. Schoch, R. R. The evolution of major temnospondyl clades: an inclusive phylogenetic analysis. *J. Syst. Palaeontol.* **11,** 67–70 (2013).

20. Schoch, R. R. & Milner, A. R. Stereospondyli. In *Handbuch der Paläoherpetologie, 3B* (ed Wellnhofer, P.), 1-203 (Verlag Dr. Friedrich Pfeil, 2000).

21. Marsicano, C. A., & Warren, A. (1998). The first Palaezoic rhytidosteid: *Trucheosaurus major* (Woodward, 1909) from the late Permian of Australia, and a reassessment of the Rhytidosteidae (Amphibia, Temnospondyli). *Bull. Nat. Hist. Mus. Lond. (Geol.)* **54,** 147–154.

22. Warren, A., & Marsicano, C. A. phylogeny of the Brachyopoidea (Temnospondyli, Stereospondyli). *J. Vert. Paleontol.* **20,** 462–483 (2000).

23. Bolt, J. R. Evolution and functional interpretation of some suture patterns in Paleozoic labyrinthodont amphibians and other lower tetrapods. *J. Paleontol* **48,** 434–458 (1974).

24. Damiani, R. A systematic revision and phylogenetic analysis of Triassic mastodonsauroids (Temnospondyli: Stereospondyli). *Zool. J. Linn. Soc.* **133,** 379–482 (2001).

25. Jupp, R., & Warren A. A. The mandibles of the Triassic temnospondyl amphibians. *Alcheringa* **10,** 99–124 (1986).
